# Nanoparticle-Based Drug Delivery Systems: An Inspiring Therapeutic Strategy for Neurodegenerative Diseases

**DOI:** 10.3390/polym15092196

**Published:** 2023-05-05

**Authors:** Linyan Duan, Xingfan Li, Rong Ji, Zhizhong Hao, Mingyue Kong, Xuejun Wen, Fangxia Guan, Shanshan Ma

**Affiliations:** 1 School of Life Sciences, Zhengzhou University, Zhengzhou 450001, China; duanlinyan4881@163.com (L.D.); lxf1217@gs.zzu.edu.cn (X.L.); jirong000@163.com (R.J.); lilachzz@icloud.com (Z.H.); 2 NHC Key Laboratory of Birth Defects Prevention, Henan Key Laboratory of Population Defects Prevention, Zhengzhou 450002, China; mykong2021@163.com; 3 Department of Chemical and Life Science Engineering, School of Engineering, Virginia Commonwealth University, Richmond, VA 23284, USA; xwen@vcu.edu; 4 Institute of Neuroscience, Zhengzhou University, Zhengzhou 450052, China

**Keywords:** neurodegenerative diseases, blood–brain barrier, nanoparticles, nanoparticle-based drug delivery systems, targeted therapy

## Abstract

Neurodegenerative diseases are common, incurable neurological disorders with high prevalence, and lead to memory, movement, language, and intelligence impairments, threatening the lives and health of patients worldwide. The blood–brain barrier (BBB), a physiological barrier between the central nervous system and peripheral blood circulation, plays an important role in maintaining the homeostasis of the intracerebral environment by strictly regulating the transport of substances between the blood and brain. Therefore, it is difficult for therapeutic drugs to penetrate the BBB and reach the brain, and this affects their efficacy. Nanoparticles (NPs) can be used as drug transport carriers and are also known as nanoparticle-based drug delivery systems (NDDSs). These systems not only increase the stability of drugs but also facilitate the crossing of drugs through the BBB and improve their efficacy. In this article, we provided an overview of the types and administration routes of NPs, highlighted the preclinical and clinical studies of NDDSs in neurodegenerative diseases, and summarized the combined therapeutic strategies in the management of neurodegenerative diseases. Finally, the prospects and challenges of NDDSs in recent basic and clinical research were also discussed. Above all, NDDSs provide an inspiring therapeutic strategy for the treatment of neurodegenerative diseases.

## 1. Introduction

The population aged 65 and over is considered to be an ageing population, and the incidence of age-related diseases, such as cancer, cardiovascular disease, and neurodegenerative diseases, increases with age. According to the Global Burden of Disease Study 2019, the global health burden of neurological diseases is approximately 8% [1]. Neurodegenerative diseases are common, incurable neurological disorders, including Alzheimer’s disease (AD), Parkinson’s disease (PD), Huntington’s disease (HD), amyotrophic lateral sclerosis (ALS), frontotemporal dementia (FTD), prion disease, and glioma, with a high incidence and can lead to memory, motor, language, and intellectual impairment. Their prevalence continues to rise globally, threatening the lives and health of patients around the world [2,3,4]. Although the clinical manifestations of these diseases are different, there are some commonalities in the pathogenesis, such as neuroinflammation, oxidative stress, neuronal cell loss, mitochondrial dysfunction, and aggregation of specific proteins [5,6,7]. Considerable progress has been made in the diagnosis and treatment of these disorders, but their outcomes have been far from satisfactory. Moreover, the advances in current treatments only relieve the symptoms of neurodegenerative diseases without actually curing them [8].

The blood–brain barrier (BBB) establishes a stable microenvironment to maintain neuronal functions [9]. Achieving sufficient drugs across the BBB is a challenge to treat central nervous system (CNS) disorders. Strategies to cross the BBB can be classified as invasive and non-invasive methods [10]. The former approach physically penetrates the BBB, whereas the latter approach bypasses the BBB without any physical breakthrough. Nanoparticles (NPs) can be used as drug transport carriers, also known as nanoparticle-based drug delivery systems (NDDSs), which not only increase the stability of drugs but also facilitate drugs crossing the BBB and improving their efficacy [11,12]. In the present article, we aimed to highlight the basic concepts and applications of nanomaterials in the preclinical and clinical studies of neurodegenerative diseases. We also made a critical analysis to discuss the prospects and challenges of NDDSs in the preclinical and clinical research of neurodegenerative diseases. To sum up, NDDS is an inspiring therapeutic strategy for the treatment of neurodegenerative diseases.

## 2. The Structure and Role of the BBB

**Figure 1:**
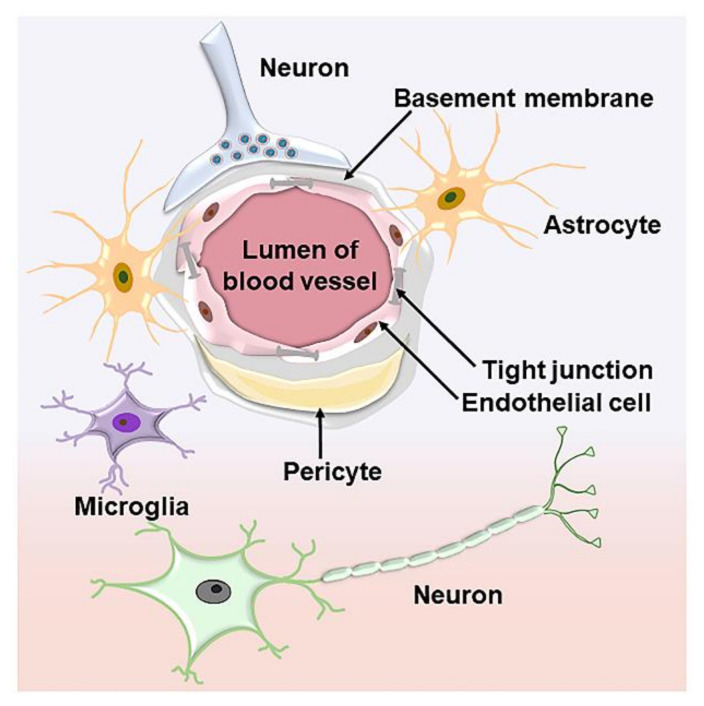
Schematic diagram of the BBB structure.

The BBB is a special cellular barrier system mainly formed by interconnected vascular endothelial cells, astrocytes, microglial cells, and basement membranes in the brain (Figure 1) [13]. The BBB plays an important role in maintaining the homeostasis of the intracerebral environment by strictly preventing most macromolecules from entering the brain and protecting the nervous system from external irritation [14]. Only very small molecules and lipophilic molecules can cross the BBB. Four different mechanisms are used by substances passing through the BBB: simple diffusion, facilitated diffusion, simple diffusion through water channels, and active translocation through protein carriers associated with active binding sites [15]. Although neurodegenerative diseases are accompanied by BBB breakdown, which may facilitate drug transport, cellular debris and microbial pathogens in the blood can also flow into the brain to initiate neurodegenerative pathways and exacerbate neurodegeneration [16]. Therefore, developing novel strategies for crossing the BBB without affecting the normal structure and function of the BBB is urgently needed.

**Figure 2:**
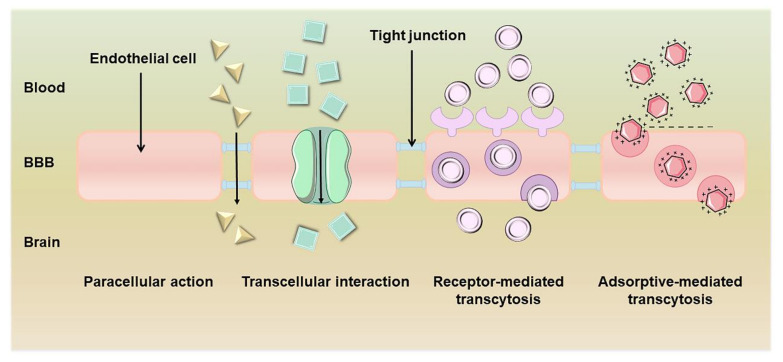
Several pathways across the blood–brain barrier.

With the breakthrough of nanotechnology, various NDDSs have been used to cross the BBB [10,17]. NPs can enhance the efficiency of BBB transport through many different pathways [18,19], such as paracellular action, transcellular interaction, receptor-mediated transcytosis, and adsorptive-mediated transcytosis (Figure 2). Paracellular action, a form of simple diffusion which reaches the intercellular space through tight connections between cells, is the preferred pathway for many water-soluble and low-lipid-soluble drugs [20]. Transcellular interaction is achieved through the distribution and facilitated diffusion of non-ionic compounds across cell membranes and consists of two forms: active and passive transport [20]. Proteins and peptides mainly enter cells via receptor-mediated transcytosis, which is active translocation through protein carriers associated with active binding sites [21]. Adsorptive-mediated transcytosis is a non-invasive technique triggered by electrostatic interactions between a positively charged substance (usually a cationic peptide or a charged molecule of a protein) and a negatively charged plasma membrane surface [22]. Thus, NDDSs represent an inspiring therapeutic strategy for neurodegenerative diseases.

## 3. Types and Administration Routes of NPs

NPs have shown great potential in overcoming the traditional evaluation of drug treatment for neurodegenerative diseases [23,24]. NPs include inorganic-based NPs, organic-based NPs, and multifunctional modified NPs [11,25]. In this section, we summarized the types and administration routes of NPs used in drug delivery systems.

### 3.1. Inorganic-Based NPs

Inorganic-based NPs are a class of NPs formed by inorganic particles with various morphologies and particle sizes ranging from 1 to 100 nm [26]. Inorganic-based NPs can not only undergo multiple surface modifications, but also bind to drug molecules in different ways, such as through electrostatic interactions, hydrophobic interactions, and covalent bonding [26]. Inorganic-based NPs mainly include metallic NPs, carbon NPs, magnetic NPs (MNPs), and nanozymes.

Gold NPs (AuNPs) have been actively studied for many years and have attracted great interest in various biomedical applications due to their biocompatibility, low toxicity, and ease of binding to biomolecules [27]. Interestingly, the surface of AuNPs can be modified by functional groups, such as thiols, amines, phosphines, and other elements, which have a high affinity for gold surfaces [28]. Silver NPs (AgNPs) are well-known for their broad-spectrum, highly effective antibacterial, antiviral, and anticancer activities, and have received special attention in biomedical fields [29]. A previous study indicated that AgNPs could affect human brain development by altering the expression of transporter proteins [30]. Another research suggested that AgNPs could induce the secretion of pro-inflammatory cytokines and the deposition of Aβ in neural cells, which may be involved in AD progression [31]. However, the effects and safety of AgNPs in brain response need further study.

In recent years, carbon nanomaterials, such as graphene, carbon nanotubes, and carbon dots (CDs), have gained widespread interest in neuroscience applications [32,33]. CDs are dispersed quasi-spherical carbon-derived NPs with good biocompatibility, ultra-small size, and tunable optical properties [34]. Different kinds of CDs and CDs-ligand conjugates have been reported to successfully penetrate the BBB [35]. For example, tryptophan CDs can cross the BBB via L-type amino acid (LAT1) endocytosis [36]. Zhou et al. (2019) found that amphiphilic yellow-emissive CDs could cross the BBB of zebrafish by passive diffusion and inhibit the deposition of Aβ in cells [37]. However, the safety of CDs remains to be further investigated.

MNPs with controllable shape, high stability, and high superparamagnetism have become a current focus of NPs research [38]. Among them, iron oxide NPs, such as Fe_3_O_4_, are the most commonly used MNPs in the biomedical field. For example, magnetic Fe_3_O_4_@SiO_2_ NPs could penetrate the BBB via the transcytosis of brain endothelial cells and magnetically mediated dragging [39], which may be a potential drug delivery system for brain diseases. Magnetite/ceria NPs were shown to alleviate oxidative stress and prevent spatial memory deficits in transgenic mice with five familial AD (5×FAD) [40]. Moreover, superparamagnetic iron oxide-loaded chitosan-coated bile body polymeric NPs could efficiently target resveratrol to the brain through the olfactory mucosa by applying an external magnetic field, and improve memory and cognitive function in lipopolysaccharide (LPS)-induced AD model mice [41]. As mentioned, MNPs could be exploited as a potential drug delivery system in regeneration medicine.

Nanozymes are enzymatically active nanomaterials that share some similarities with natural enzymes in terms of overall size, shape, and surface charge, but are more resistant to harsh environments and can adapt to wide pH range and temperature variations [42]. A novel amino-nanozyme based on boehmite NPs functionalized with tetra-azapyridinophane could cross the BBB, scavenge mitochondrial reactive oxygen species, and disaggregate mutant Huntington deposits [43].

In conclusion, inorganic-based NPs have been widely used because of their small size, controllable shape, and high stability. However, due to their potential neurotoxic effects, inorganic-based nanoparticles need to be manufactured and used with a focus on the associated toxic effects in order to avoid serious effects on humans and other organisms.

### 3.2. Organic-Based NPs

Organic-based NPs are biodegradable and can trigger drug release upon cellular uptake and internalization, resulting in prolonged therapeutic effects [44]. Organic-based NPs can be prepared in a variety of ways, including solvent displacement, chemical reduction in solution, ionic association, and photochemical methods [45]. The major organic-based NPs can be classified into three categories: lipid-based NPs [46], polymeric NPs, and natural NPs [47,48].

Lipid-based NPs can be divided into liposomes and lipid NPs (LNPs) [46]. Liposomes are considered to be the earliest generation of lipid NPs and the first nanomedicine delivery platform to successfully move from concept to clinical application. The next generation of LNPs, including solid lipid NPs and nanostructured lipid NPs, exhibit more complex internal structures and greater physical stability. Due to their biocompatibility and the presence of hydrophilic and hydrophobic components, liposomes and LNPs have a great affinity for targeting the BBB and can encapsulate both water-soluble and lipophilic drugs, thereby increasing their bioavailability in vivo. A previous study demonstrated that repeated intraperitoneal injections of liposomes containing phosphatidic acid and cardiolipin reduced Aβ levels in the plasma and brain of APP/PS1 transgenic mice [49]. Thöle M et al. (2002) confirmed that solid lipid NPs with appropriate ligands can be taken up by intact brain capillaries to ensure the penetration of the BBB and can be used to deliver drugs to the brain [50]. Thus, lipid-based NPs can increase drug solubility, improve drug bioavailability and therapeutic efficacy, and reduce the therapeutic dose of drugs.

Different types of polymers, such as poly(lactic-co-glycolic acid) (PLGA), polyethylene glycol (PEG), hyaluronic acid, chitosan, and alginate, are also used to synthesize NPs for the delivery of drugs, such as polysaccharides, proteins, amino acids, and polyesters [51]. Polymeric NPs, which combine the excellent biochemical properties of polymers with the size effect of NPs, have attracted extensive attention due to their good biodegradability and nontoxicity. In addition, polymeric NPs facilitate multiple modifications and have a high capacity, which may be a good choice for drug delivery to the CNS.

Natural NPs including cell membranes and cell-derived extracellular vesicles (EVs) have been explored as alternatives for brain drug delivery because of their low toxicity, sustainability, and biodegradability [48,52]. Red blood cell membrane-disguised human serum albumin NPs loaded with curcumin could alleviate the symptoms of AD by reducing mitochondrial oxidative stress and inhibiting neuronal death [53]. In addition to synthetic nanocarriers, cell-derived EVs-based carrier systems have attracted considerable interest. EVs are heterogeneous lipid-bound NPs composed of many lipids and membrane proteins [54]. Some of these components have an inherent tissue-homing ability, whereas others minimize non-specific interactions [55,56]. Morad et al. (2019) demonstrated that tumor-derived EVs penetrated the BBB through transcellular interactions [57]. Food industry by-product-derived EVs showed good biocompatibility, high ability to pass through the BBB, and excellent oral bioavailability [58], representing a next-generation drug delivery platform for biomedical applications. Overall, these studies indicate that organic-based NPs have a wide range of promising applications in the treatment of neurodegenerative diseases and thus will certainly be explored further.

In summary, organic-based NPs have many advantages, such as better biocompatibility, structural versatility, and great scope for chemical modifications. These advantages can also give nanoparticles a variety of functions, such as targeting and stimulus response, but the complexity of their production and preparation limits their further use in brain drug delivery.

### 3.3. Multifunctionally Modified NPs

There is a great scope for chemical modifications of NPs that can be used to confer a variety of functions to NPs, such as targeting and stimuli-responsive functions [59,60]. In neurodegenerative diseases, commonly used modifications are cell-penetrating peptides (CPPs), which are short peptide sequences (5–30 amino acids) to help impermeable substances cross the BBB [61]. Functionally modified polymeric NPs with CPPs can improve drug delivery efficiency and therapeutic efficacy. CPPs can be simply classified into three major groups: cationic, amphiphilic, and hydrophobic peptides [62]. The natural CPPs mainly include Tat (GRKKRRQRRR) derived from human immunodeficiency virus (HIV) [63], R8 (RRRRRRRR) [64], penetratin (PEN, RQIKIWFQNRRMKWKK) [65], etc. In addition, cystatin-dense peptide binds to the transferrin receptor (TfR) allowing for the easier delivery of targeted drugs to the CNS [66]. Liposomal NPs bifunctionally modified with mannose and CPPs exhibited higher transport efficiency across the BBB [67]. Thus, multifunctionally modified NPs can be used as potential drug delivery systems due to their high targeting and high transmembrane efficiency, however, their preparation process faces certain challenges.

### 3.4. Administration Routes of NPs

The common administration routes for NPs delivery include intravenous (from blood to the brain), nasal (from nose to the brain), and oral (from gut to the brain). Intravenous administration can avoid first-pass metabolism and allow for accurately controlling blood concentrations, but it may cause systemic infections and has a low drug-targeting ability. However, the Dedrick model predicts that as compared to intravenous administration, intra-arterial administration may be effective with low local blood flow, high local extraction, and systemic clearance [68]. Additionally, altering the properties of candidate drugs and physiological variables can have profound effects on regional deposition after intra-arterial injections [69]. Intranasal administration is a non-invasive method with the advantages of less trauma, fewer adverse reactions, and the prevention of gastrointestinal reactions and first-pass effects [70]. However, the effective utilization of the drug is significantly reduced due to mucociliary clearance and enzymatic degradation [71]. The digestive tract appears to be an alternative pathway for receiving NPs. A recent study showed very low TiO_2_ concentrations in the intestine of rats after the intra-esophageal infusion of TiO_2_ NPs, but TiO_2_ NPs were faintly but clearly detectable in the brain [72]. However, this study was not able to accurately determine the cellular location and the exact intracerebral translocation pathway. Collectively, administration routes of NPs require careful design to improve their delivery efficiency in crossing the BBB and targeting the brain.

## 4. NPs-Based Drug Delivery Systems

**Figure 3:**
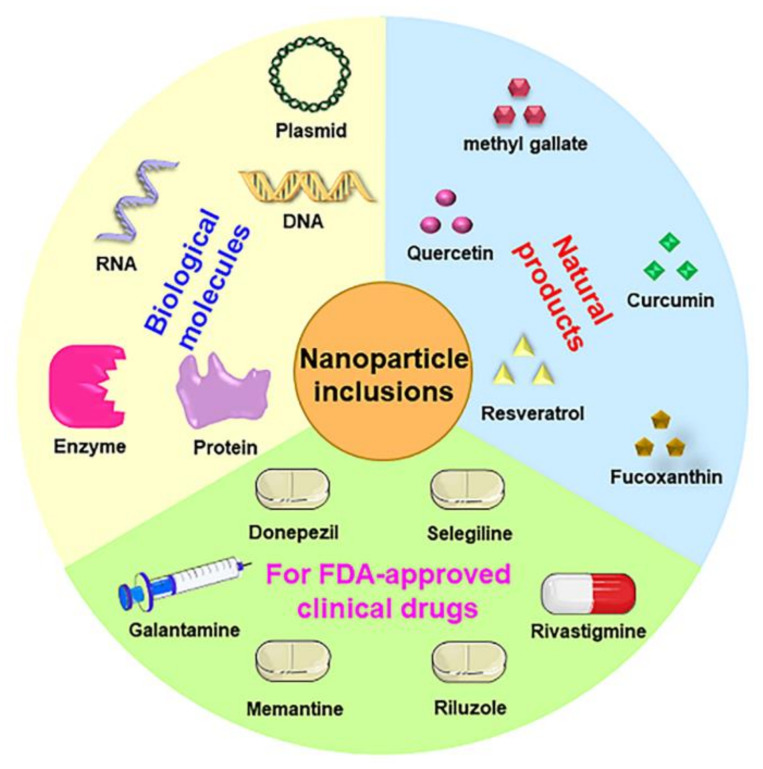
Application of NDDSs loaded with different substances in the treatment of neurodegenerative diseases.

NDDSs can encapsulate different drugs, such as biological molecules, natural products, and Food and Drug Administration (FDA)-approved clinical drugs (Figure 3), deliver them efficiently to the target location, and modulate the pharmacokinetic properties, efficacy, and toxicity of the drugs [73]. As such, NDDSs are an inspiring drug delivery strategy and have received widespread interest in the treatment of cancers and neurodegenerative diseases [74].

### 4.1. Biological Molecules

NDDSs can improve the utilization and delivery of biomolecules, which have strict molecular recognition functions. The common biological molecules encapsulated in NDDSs mainly include DNA, siRNA, proteins, enzymes, amino acids, and peptides.

Liposomal NPs dual-modified with transferrin (Tf) and a PEN-encasing nerve growth factor (NGF) plasmid were shown to efficiently cross the BBB and reduce Aβ accumulation in AD mice [75]. A liposomal apolipoprotein E2 (ApoE2) gene delivery system was shown to evade the BBB for the effective treatment of AD [76]. In addition, β-site APP cleavage enzyme 1 (BACE1) could cleave the amyloid precursor protein to induce the accumulation of Aβ [77]. Therefore, reducing BACE1 activity is considered a potential therapy for AD. Zhou et al. (2020) found that a glycosylated triple-interaction-stabilized siBACE1 nanodrug could efficiently penetrate the BBB and ameliorate AD-like pathology in APP/PS1 mice [78]. A peptide delivery nanoconjugate (Ab peptide, p-Nrf2 peptide, and PEG-based biomimetic dendrimer) could effectively span the BBB, eliminate reactive oxygen species (ROS), and release p-Nrf2, which had a synergistic effect on restoring cellular antioxidant capacity and alleviating glial cell activation [79]. Furthermore, sodium hyaluronate NPs could be used to carry recombinant neuroglobins to the brain, achieving neuroprotective effects in neurological diseases [80]. Taken together, these findings reveal that an NDDS-mediated biological molecule delivery is a potential treatment strategy for neurodegenerative diseases. However, it is important to note that the dosage of biomolecules needs further quantitative design, and excess biomolecules may trigger inflammation or other immune responses.

### 4.2. Natural Products

Natural products, such as flavonoids, terpenoids, saponins, and polyphenols, are a class of ingredients or metabolites derived from plants or microorganisms with specific functions and are widely used in medical fields [81]. However, these extracts are usually characterized by low water solubility, low bioavailability, and short biological half-life, which, in turn, limit their clinical applications [82]. Therefore, their bioavailability should be improved through the use of NDDSs for further translational studies.

Quercetin is a natural phytotherapeutic compound widely found in fruits and vegetables with multifunctional bioprotective effects, such as anti-neurodegenerative effects [83]. Quercetin-modified gold-palladium NPs had high BBB permeability and accelerated the clearance of Aβ [84]. Yang et al. (2018) found that curcumin-loaded chitosan-bovine serum albumin NPs enhanced the phagocytosis of Aβ42 and modulated macrophage polarization in AD mice [85]. In addition, oral administrations of resveratrol-selenium-peptide NPs alleviated AD-like pathogenesis by inhibiting Aβ aggregation and regulating gut microbiota [86]. Resveratrol encapsulated in solid lipid NPs successfully improved the bioavailability and distribution of resveratrol in the brain and targeted the BBB [87]. Above all, NPs loaded with natural products represent a potentially viable approach to enhance their bioavailability and thus improve their delivery in the brain and contribute to the treatment of neurodegenerative diseases. Despite the many promising advantages, further studies are needed to investigate their stability in living organisms and the dissociation rate of the nanosystems.

### 4.3. FDA-Approved Clinical Drugs

Many drugs, such as memantine [44], donepezil [44], galantamine [88], and riluzole [89], have been approved by the FDA for the treatment of neurological diseases. However, these drugs have limited efficacy in treating or slowing disease progression with side effects such as functionality, gastrointestinal disorders, and headaches [90]. Therefore, novel drug delivery systems should be developed to enhance the local therapeutic effect of these drugs and reduce their side effects.

Donepezil, an acetylcholinesterase (AChE) inhibitor, is commonly used to treat moderate AD, but conventional donepezil treatment dictates that it is taken daily to maintain efficacy [91]. Donepezil-loaded cholesterol-modified pullulan NPs exhibited sustained drug release and significant brain targeting, superior to that of free donepezil [92]. Another study showed that PLGA-b-PEG NPs loaded with donepezil exhibited a controlled release profile and high BBB penetration to induce the destabilization of amyloid fibrils [93]. Galantamine (GAL) has the dual effects of inhibiting cholinesterase activity and modulating nicotinic acetylcholine receptor activity. PLGA NPs loaded with GAL had high encapsulation efficiency and sustained drug release to maintain the pharmacological activity of GAL and produce long-term therapeutic effects in neurodegenerative diseases [88]. Memantine, an N-methyl-D-aspartate receptor antagonist, is used to treat moderately severe to severe Alzheimer’s-type dementia [94]. PLGA-pegylated NPs loaded with memantine were able to cross the BBB, reduce Aβ plaques, and were more effective than memantine alone in AD mice [95]. Selegiline is a well-known anti-Parkinson drug with poor oral bioavailability and safety. Chitosan NPs loaded with selegiline exerted better therapeutic efficacy than selegiline treatment alone [96]. Riluzole is an effective neuroprotective agent for the treatment of severe motor neuron disorders in ALS [89] with poor water solubility. Chitosan-conjugated NPs could carry large amounts of riluzole across the BBB, producing significant neuroprotective effects, even at very low concentrations, in a model of cerebral ischemia [97]. Collectively, NDDSs encapsulated with FDA-approved drugs demonstrate excellent therapeutic value and represent optimized therapeutic strategies for the treatment of neurodegenerative diseases, but there are currently few FDA-approved clinical drugs, and NPs loaded with FDA-approved clinical drugs will have to be studied in the future with more attention to their particle aggregation and toxicity.

## 5. Application of NPs in Neurodegenerative Diseases

As mentioned above, NPs and NDDSs provide effective tools and opportunities to penetrate the BBB and improve drug efficacy in the management of neurodegenerative diseases. The preclinical applications of NPs in AD, PD, HD, ALS, FTD, prion disease, and glioblastoma (GBM) over the past five years are shown in Table 1.

### 5.1. Alzheimer’s Disease

AD is a chronic neurodegenerative disease with high prevalence and incremental cognitive deficits [114]. However, FDA-approved drugs, such as donepezil and memantine, can only ameliorate AD symptoms to a certain extent and cannot completely cure AD [115,116]. The important factors driving the occurrence and development of AD include Aβ accumulation, tau phosphorylation, neuroinflammation, metabolic dysfunction, and mitochondrial dysfunction, such as mitochondrial autophagy and mitochondrial protein deposition damage [117,118,119,120,121]. Therefore, the inhibition or blockade of these pathogenic mechanisms is the key to treating AD. Studies showed that AuNPs prevented cognitive impairment, oxidative stress, and neuroinflammation in a rat model of AD [122]. In addition, AuNPs functionalized by TPM (a maize-derived tetrapeptide) could modulate animal behavior, oxidative stress, and the cholinergic system to enhance neuroprotective effects in AD mice [99]. An aqueous extract of seaweed leaves used as a AgNP-stabilizing agent successfully prevented streptozotocin-induced recognition and spatial memory deficits in a sporadic AD rat model [100]. Magnetite/ceria NPs were shown to alleviate oxidative stress and prevent spatial memory deficits in 5×FAD mice [40]. Guo et al. (2021) demonstrated that multifunctional selenium quantum dots not only effectively inhibited Aβ aggregation but also reduced oxidative stress and restored mitochondrial function [101]. In addition, Yang et al. (2021) showed that PLGA-PEG-loaded fucoxanthin NPs penetrated the BBB and sustainedly released fucoxanthin to prevent cognitive impairments in Aβ-induced AD mice with greater efficacy than that of free fucoxanthin [102]. Recent reports indicated that self-fluorescent tryptophan NPs could alleviate cognitive deficits and inhibit Aβ42 oligomerization in the brain of AD rats [98]. These findings suggest that NPs may be an effective treatment strategy for AD. However, key issues such as the metabolism and toxicity of metal ions in vivo should be considered.

### 5.2. Parkinson’s Disease

PD is usually accompanied by the necrosis of dopaminergic neurons, the aggregation of α-synuclein (α-syn), and decreases in dopamine [123]. The existing therapeutic strategies for PD mainly focus on the loss of dopamine and dopaminergic function, which have high rate of side effects and lack long-term efficacy [124,125]. Cerium oxide (CeO_2_) NPs mitigated the amyloid formation of α-syn and associated cytotoxicity [126]. CeO_2_ NPs were shown to counteract α-syn-induced mitochondrial dysfunction and decrease ROS production in a yeast model of PD [127], while Mn_3_O_4_ NPs successfully reduced α-syn levels in the cerebrospinal fluid of PD mice, improved their cognitive function, and exhibited good biodegradability [104]. AuNPs reversed the PD symptoms induced by alkaline reserpine in C57BL/6 mice, and alleviated neuronal cell death induced by reserpine [103]. TP10-dopamine NPs synthesized by coupling dopamine to the cell-penetrating peptide TP10 showed a high affinity for dopamine D1 and D2 receptors and had obvious resistance to PD activity [106]. PLGA/puerarin NPs exhibited neuroprotective effects by ameliorating 1-Methyl-4-phenyl-1,2,3,6-tetrahydropyridine (MPTP)-induced behavioral deficits and dopamine depletion symptoms in PD mice [105]. PEG/chitosan NPs loaded with ellagic acid attenuated rotenone-induced cytotoxicity in a PD model [128]. Thus, these findings indicate the potential of NPs as a new therapy for PD.

### 5.3. Huntington’s Disease

HD is an incurable neurodegenerative disease caused by the repeated expansion of the CAG trinucleotide sequence in the first exon of the Huntington (HTT) gene [129]. The drugs with formal indications for treating HD patients are tetrabenazine and deutetrabenazine, which are associated with adverse effects [130]. Therefore, revolutionary therapies are under development for the treatment of HD. Poly(alginate) NPs were shown to reduce the cytotoxicity caused by amyloid/polyglutamine aggregation and prevent polyglutamine aggregation in the brain of HD model mice [131]. Polyglutamine-specific AuNPs decorated with amphiphilic peptides and polyethyleneimine on their surfaces can be transported to the brain, dissociating mutant HTT and ameliorating functional deterioration in the HD drosophila larva model [107]. In addition, self-assembled polymeric NPs based on epigallocatechin-3-gallate (EGCG) could protect neuronal cells from the toxic effects of extracellular Aβ or intracellular mutant HTT protein aggregates [132]. Selenium NPs reduced neuronal death and alleviated behavioral deficits by inhibiting oxidative stress and the aggregation of HTT proteins in transgenic HD models of *Caenorhabditis elegans* [108]. NPs loaded with cholesterol improved synaptic and cognitive function in HD mice [109]. Taken together, these encouraging results confirm that NPs and NDDSs are promising therapeutic strategies for the effective management in the prevention and treatment of HD.

### 5.4. Amyotrophic Lateral Sclerosis

ALS is a progressive and fatal neurodegenerative disease due to the degeneration of motor neurons, with no effective treatment [133,134,135]. Recent studies showed that cerium oxide NPs or mesoporous silica NPs loaded with trophic factor peptide mimics preserved muscle function and prolonged the lifespan of ALS mice model [111,136]. Moreover, AuNPs loaded with FM19G11 (a hypoxia-inducible factor) may be a novel tailored approach to delaying ALS progression, as shown by the enhancement of proliferation and the self-renewal of ependymal stem progenitor cells in ALS mice [110]. The dysregulation of the retinoic acid signaling pathway also plays an important role in the development of ALS [137]. Retinoid-activating NPs improved motor performance, prolonged lifespan, and played a neuroprotective role in the SOD1G93A mouse model of ALS [112]. Moreover, lactoferrin-functionalized lipid NPs facilitated the transport of riluzole across the BBB by interacting with lactoferrin receptors expressed on brain endothelium in ALS treatment [138]. Collectively, these findings highlight the advantages and potential of NPs or NDDSs in the treatment of ALS.

### 5.5. Frontotemporal Dementia, Prion Disease, and Glioblastoma

FTD is a heritable dementia syndrome accompanied by frontotemporal lobe atrophy, personality changes, and cognitive impairment [139]. Prion disease is a neurodegenerative disease of humans and animals, in which prion protein plays a vital role in pathogenesis [140]. GBM is a kind of primary brain cancer, belonging to a heterogeneous collection of brain tumors [141]. There is currently no effective therapy for these diseases and new drug delivery technologies that can bypass the BBB need to be developed [140,141]. Binyamin et al. (2015) found that a nanodrop formulation of pomegranate seed oil (PSO) could greatly reduce demyelination and lipid oxidation in the brains of diseased animals, suggesting that nano-PSO may also be beneficial in the prevention and treatment of hereditary prion disease [142]. EI Moustaine et al. (2008) created amyloid nanofibrils and NPs from recombinant prion protein under high pressure, which provided an understanding of the misfolding of prion protein into amyloid [143]. Lin et al. (2016) developed BBB-penetrating albumin NPs for the dual-drug delivery of paclitaxel and fenretinide, and these albumin NPs exhibited improved treatment outcomes in glioma models with reduced toxic side effects [144]. Bioreducible polymeric NPs containing multiplexed cancer stem cell-regulating miRNAs significantly inhibited glioblastoma growth and prolonged survival [113], demonstrating the promise of using nanotechnology in combination with cancer stem cell-inhibiting miRNAs for treating GBM. In conclusion, nanomedicine-based therapeutic strategies play an important role in slowing down the progression of FTD, prion disease, and GBM.

## 6. Combined Therapeutic Strategies

Numerous studies have shown the possibility of NDDSs combined with other strategies in treating neurodegenerative diseases. (1) NDDSs combined with FDA-approved drugs. NDDSs have been combined with FDA drugs such as memantine [95], donepezil [92], galanthamine [88], and riluzole [97] to improve the efficacy of the drugs in treating neurodegenerative diseases. (2) NDDSs combined with immunotherapy [145]. Kuang et al. (2018) designed iRGD (a peptide that could penetrate the tumor tissue and target the tumor cells)-modified silica NPs to simultaneously deliver doxorubicin and the immune checkpoint inhibitor 1-methyltryptophan [146]. iRGD was able to guide the penetration of NPs through the BBB and enhance drug accumulation in gliomas. At the same time, NPs induced anti-tumor immune responses and modulated the immunosuppressive microenvironment to significantly prolong moderate survival [146]. (3) NDDSs combined with phototherapy. Phototherapy is a promising non-invasive strategy for cancer treatment. Yu et al. (2018) designed composite NPs synergistically loaded with a photosensitizer and a therapeutic drug that could circulate in the blood and actively target glioma cells for a prolonged period [147]. (4) NDDSs combined with focused ultrasound (FUS). FUS is a reversible way to open the BBB using acoustic energy in targeted brain regions [148]. FUS combined with the microvesicles is considered as the only technique for the non-invasive and reversible destruction of the BBB, allowing molecules to cross into the brain parenchyma [149]. Mead et al. (2017) designed a two-pronged treatment strategy in which FUS opened the BBB in targeted areas and mediated the intracerebral delivery of glial cell-line derived neurotrophic factor (GDNF) loaded brain-penetrating NPs. This combined technique was able to induce widespread and targeted GDNF transgene expression in the brain following the systemic administration to LPS-treated rats, thereby reversing PD behavioral abnormalities in a very safe and effective manner [150]. Thus, many combined therapeutic strategies have been developed and showed enhanced therapeutic effects in animal models. However, they are rarely used in clinical trials. Therefore, more in-depth research is needed to develop an optimal combined treatment strategy.

## 7. Clinical Trials

Although many in vivo and in vitro studies have been conducted to investigate the efficiency and therapeutic potential of NPs and NDDSs, only a few clinical trials (https://clinicaltrials.gov/, accessed on 11 November 2022) have involved the use of nanocarriers to target neurodegenerative diseases (Table 2). These clinical studies mainly aimed to evaluate the safety, tolerability, metabolic effects, and efficacy of NPs alone or in combination with other therapies for the treatment of AD, PD, ALS, and glioma, and clarify the potential of using NPs in the diagnosis and clinical management of neurodegenerative diseases. Although the results of these clinical studies have not been published or may not be satisfactory, this has not stopped further clinical research efforts.

## 8. Prospects and Challenges

Many preclinical and clinical studies have been conducted on neurodegenerative diseases in the last few decades, but drug efficacy is still unsatisfactory due to the presence of the BBB. NDDSs increase the efficacy of drug delivery to the brain and have great potential for the treatment of neurodegenerative diseases. Unfortunately, no effective therapeutic nanotherapeutics have been successfully introduced to the clinical market. Thus, the preclinical research and clinical translation of nanotherapeutics require further in-depth exploration to solve the associated issues, including the biocompatibility and safety of nanomedicines, their interaction with the biological environment, and regulatory requirements. The main challenges and difficulties that need to be considered and addressed before entering clinical trials include the followings. (1) The safety of long-term use must be evaluated. Due to the unique properties of NPs, conventional toxicological evaluations are usually unable to accurately assess the safety of nanomedicines. Further, the available in vivo and clinical data on the neurotoxicity of NDDSs are still scarce, especially in the brains of elderly people who have reduced homeostatic capacity [153]. (2) The exact distribution of these NDDSs in the brain must be investigated. The common delivery methods for NPs include intravenous (from blood to the brain), oral (from gut to the brain), and nasal (from nose to the brain) [154]. However, NDDSs and existing technologies require further careful design to improve their delivery efficiency in crossing the BBB and targeting the brain. (3) Most of the studied animal models are based on non-primate models with limited reliability [155]. Collectively, it is urgent to develop suitable safety assays for nanomedicines to clarify the mechanisms and targeting of different drug delivery routes to the CNS and conduct more preclinical studies in primate models to translate more nanomedicines to clinical applications.

## 9. Conclusions

The boom in nanotechnology offers great promise for the development of nanocarriers that help to overcome the BBB. Although much work on the clinical applications of NDDSs must be done, current studies demonstrated their great potential as an inspiring therapeutic strategy in the treatment of neurodegenerative diseases. As research continues, we believe that NDDSs will bring great opportunities and broader prospects for the treatment of neurodegenerative diseases.

## Figures and Tables

**Table 1:** Preclinical studies of NPs in neurodegenerative diseases over the past 5 years.

Nanomaterials	Administration Route	Animal Models	Effectiveness	Ref.
Self-fluorescent solo tryptophan nanoparticles	Intraventricular administration	Streptozotocin induced AD model rat	Attenuates cognitive deficits and inhibits Aβ_42_ oligomerization	[98]
Gold nanoparticles	Subcutaneous administration	D-galactose and aluminum chloride induced AD model mice	Modulate animal behavior, oxidative stress, neurotransmitter levels, and cholinergic system	[99]
Silver nanoparticles	Intraventricular administration	Streptozotocin induced AD model rat	Prevent recognition and spatial memory impairment	[100]
Selenium quantum dots	Intravenous administration	Aβ_1–42_ induced AD model mice	Inhibits Aβ aggregation, and reduces oxidative stress	[101]
Magnetite/ceria nanoparticles	Intravenous administration	5×FAD transgenic AD model mice	Reduce Aβ levels, and prevent memory deficits	[40]
PLGA-PEG nanoparticles	Intravenous administration	Aβ oligomers induced AD model mice	Prevention of cognitive impairment induced by Aβ	[102]
Gold nanoparticles	Intraperitoneal administration	Alkaline reserpine induced PD model mice	Reduce secondary neurodegenerative processes and neuronal cell death	[103]
Mn_3_O_4_ nanoparticles	Striatum administration	MPTP-induced PD model mice	Decrease the content of α-syn in cerebrospinal fluid	[104]
Six-armed star-shaped PLGA nanoparticles	Oral administration	MPTP-induced PD model mice	Reduce dopamine depletion in MPTP-mediated neurotoxicity in mice	[105]
TP10-dopamine nanoparticles	Intravenous administration	MPTP-induced PD model mice	High affinity for dopamine D1 and D2 receptors and obvious resistance to PD activity	[106]
Gold nanoparticles	-	HD fruit fly larva model	Improved motor performance and longevity	[107]
Selenium nanoparticles	-	HD *Caenorhabditis elegans* model	Reduces neuron death, and alleviates oxidative stress	[108]
PLGA/cholesterol nanoparticles	Intraperitoneal administration	R6/2 transgenic HD model mice	Enhance biosynthesis of endogenous cholesterol, prevented cognitive decline	[109]
Gold nanoparticles	-	SOD1-G93A transgenic ALS model mice	Promote the self-renewal and proliferation of epSPC	[110]
Silica nanoparticles	Intrathecal administration	SOD1-G93A transgenic ALS model mice	Delay disease progression and increased survival in mice	[111]
PLA-PEG nanoparticles	Intravenous administration	SOD1-G93A transgenic ALS model mice	Improve motor performance and longevity	[112]
Poly(beta-amino ester) nanoparticles	Intratumoral administration	Mice with GBM1A glioma xenograft	Inhibit glioblastoma growth and prolong survival	[113]

**Table 2:** Recent clinical trials with NPs for the treatment of neurodegenerative diseases.

Status	Study Title	Conditions	Interventions	Phase	Identifier
Not yet recruiting	Study of APH-1105 in patients with mild to moderate Alzheimer’s disease	Dementia Alzheimer Disease 1 Alzheimer Disease 2 Alzheimer Disease 3	Drug: APH-1105 Other: Placebo	Phase 2	NCT03806478
Completed	31P-MRS imaging to assess the effects of CNM-Au8 on impaired neuronal redox state in Parkinson’s disease (REPAIR-PD)	Parkinson’s Disease	Drug: Gold Nanocrystals	Phase 2	NCT03815916
Completed	Exploratory study using nanotechnology to Detect Biomarkers of Parkinson’s Disease From Exhaled Breath	Parkinson’s Disease Parkinsonism	Other: collection of exhaled breath	-	NCT01246336
Completed	Therapeutic nanocatalysis to slow disease progression of Amyotrophic Lateral Sclerosis (ALS) (RESCUE-ALS)	Amyotrophic Lateral Sclerosis	Drug: CNM-Au8 Drug: Placebo	Phase 2	NCT04098406 [151]
Withdrawn (study execution discontinued at this time)	31P-MRS imaging to assess the effects of CNM-Au8 on impaired neuronal redox state in Amyotrophic Lateral Sclerosis (REPAIR-ALS) (REPAIR-ALS)	Amyotrophic Lateral Sclerosis	Drug: Gold Nanocrystals	Phase 2	NCT03843710
Completed	A Phase I trial of nanoliposomal CPT-11 (NL CPT-11) in patients with recurrent high-grade gliomas	Glioblastoma Gliosarcoma Anaplastic Astrocytoma Anaplastic Oligodendroglioma	Drug: Nanoliposomal CPT-11	Phase 1	NCT00734682
Recruiting	AGuIX nanoparticles with radiotherapy plus concomitant Temozolomide in the treatment of newly diagnosed glioblastoma (NANO-GBM)	Glioblastoma	Drug: Polysiloxane Gd-Chelates based nanoparticles (AGuIX) Radiation: radiotherapy Drug: Temozolomide	Phase 1 Phase 2	NCT04881032 [152]

## Data Availability

Not applicable.

## Abbreviations

AD, Alzheimer’s disease; PD, Parkinson’s disease; HD, Huntington’s disease; ALS, amyotrophic lateral sclerosis; FTD, frontotemporal dementia; GBM, glioblastoma; BBB, blood–brain barrier; CNS, central nervous system; NPs, nanoparticles; NDDSs, nanoparticle-based drug delivery systems; AuNPs, gold nanoparticles; AgNPs, silver NPs; Aβ, amyloid-β; CDs, carbon dots; LAT1, L-type amino acid transporter 1; LPS, lipopolysaccharide; GO, graphene oxide; EVs, extracellular-vesicles; LNPs, lipid nanoparticles; PLGA, poly(lactic-co-glycolic acid); PEG, polyethylene glycol; CPPs, cell-penetrating peptides; HIV, human immunodeficiency virus; PEN, penetratin; TfR, transferrin receptor; FDA, Food and Drug Administration; Tf, transferrin; NGF, nerve growth factor; ApoE2, apolipoprotein E2; BACE1, β-site APP cleavage enzyme 1; ROS, reactive oxygen species; AChE, acetylcholinesterase; GAL, galantamine; PSO, pomegranate seed oil; TPM, a maize-derived tetrapeptide; CeO_2_, cerium oxide; MPTP, 1-Methyl-4-phenyl-1,2,3,6-tetrahydropyridine; α-syn, α-synuclein; HTT, Huntington; EGCG, epigallocatechin-3-gallate; FUS, focused ultrasound; GDNF, glial cell line-derived neurotrophic factor.
